# Cranial Ultrasonography—Standards in Diagnosis of Intraventricular Hemorrhage and Ventricular Dilatation in Premature Neonates

**DOI:** 10.3390/children12060768

**Published:** 2025-06-13

**Authors:** Adriana Mihaela Dan, Diana Iulia Vasilescu, Ion Dragomir, Sorin Liviu Vasilescu, Diana Voicu, Monica Mihaela Cîrstoiu

**Affiliations:** 1 Faculty of Medicine, “Carol Davila” University of Medicine and Pharmacy Bucharest, 050474 Bucharest, Romania; adriana.dan@umfcd.ro (A.M.D.); diana.voicu@umfcd.ro (D.V.); monica.cirstoiu@umfcd.ro (M.M.C.); 2 Department of Neonatology, Emergency University Hospital Bucharest, 050098 Bucharest, Romania; i.dragomirion@gmail.com; 3 Department of Obstetrics and Gynecology, Emergency University Hospital Bucharest, 050098 Bucharest, Romania; drsorinvasilescu@gmail.com

**Keywords:** intraventricular hemorrhage, risk factors, neurologic outcome, cranial ultrasound, posthemorrhagic ventriculomegaly

## Abstract

Intraventricular hemorrhage (IVH) is a common complication encountered in extremely-low-birth-weight (ELBW) and very-low-birth-weight (VLBW) premature babies. The neurologic outcome of these patients is influenced by the magnitude of the hemorrhagic process that damages the involved anatomic structures but also by the impaired circulation of cerebrospinal fluid (CSF) through the ventricular system, leading to posthemorrhagic ventriculomegaly (PHVM). Cranial ultrasound (CUS) performed by neonatologists (point-of-care ultrasound—POCUS) facilitates the early diagnosis of IVH and PHVM and can objectively quantify structural alterations. Our aim was to identify the best sonographic criteria to follow-up with ventricular dilatation and predict the need for neurosurgery and neurologic deterioration. We performed a literature review in search of the most relevant ventricular measurements considered by neurosurgeons, neonatologists, and pediatric neurologists to reflect the risk of white matter injury and high intracranial pressure (HIP), thus anticipating neurologic developmental impairment (NDI). The tridimensional picture of ventricular dilatation is best captured if more than one index (ventricular index and anterior horn width) or ratio (Evans ratio, fronto-occipital horn ratio, and fronto-temporal horn ratio) is used. **Conclusions**: If performed using the correct protocol, serially and comprehensively, CUS is an indispensable tool for the diagnosis and follow-up of neurologic complications of preterm babies, and it can make a difference in guiding adequate intervention and improving long-term developmental outcomes.

## 1. Introduction

### 1.1. Intraventricular Hemorrhage—Still a Common Complication of Extreme Prematurity

Intraventricular hemorrhage (IVH) remains a severe complication encountered in extremely-low-birth-weight (ELBW) and very-low-birth-weight (LBW) premature babies, despite better acknowledgement of determining factors and progress that has been made in the care of this category of patients. The global incidence of IVH is reported to be 20–38% in premature infants < 28 weeks gestational age and 15% in infants between 28 and 32 weeks [1], and depending on cumulated risk factors, it can exhibit different grades of severity. Grade I represents a hemorrhage confined to the germinal matrix, grade II describes small amounts of blood moving into the lateral ventricle, and grade III is defined by the enlargement of the lateral ventricle by the presence of an echogenic clot that occupies more than 50% of the ventricular volume (Papile classification) [2,3]. Intraparenchymal echogenicity had been considered by Papile a grade IV hemorrhage, resulting from the disruption of the ventricular wall and extension of the IVH, but it is nowadays acknowledged as a periventricular hemorrhagic infarction (PVHI), the result of venous congestion followed by hemorrhage [1,4]. Grade III IVH and PVHI are more common in infants under 28 weeks (10–15%) [1]. In many medical centers, IVH is the most important complication of prematurity related to a higher mortality of preterm infants, inversely proportionally correlated with low gestational age and birth weight, with values up to 50–60% for grade III IVH and PVHI and 20% if the IVH remains of grade I or II [1,4,5].

Grade I or II IVH can be completely resolved, while for grade III, there is a slow or rapid progression to posthemorrhagic ventriculomegaly (PHVM), usually between 7 and 14 days after the acute event [6,7]. The extent of the hemorrhage is directly proportional to the risk of developing PHVM. According to various studies, 65–70% of newborns with severe IVH have a slow progression and stabilization or even regression of ventricular volume, while 30–35% will display a rapid progression over days or weeks [6]. The resorption of the blood clots takes weeks, with residual lesions, these being small cysts, detected in the germinal matrix (in grade I or II hemorrhages) or in one or more porencephalic paraventricular cysts, distinct or confluent to the ipsilateral ventricle, in case of PHVI [1,8].

### 1.2. Posthemorrhagic Ventriculomegaly (PHVM)—Different Pathogenic Mechanisms

Newborns with severe IVH have a 30–50% risk of developing PHVM [4] due to disturbances in either the production or reabsorption process of CSF [6,7]. If drainage is impaired, the condition is also called obstructive hydrocephalus [4], and CUS identifies visible clots in the aqueduct of Sylvius, foramen of Luschka, or foramen of Magendie [4,9]; in some cases, obstruction may be caused by multiple microthrombi, or by post-inflammatory scarring at the arachnoid granulation (obliterative arachnoiditis), that cannot be detected by ultrasonographic examination [10,11,12]. The overproduction of CSF causes communicating hydrocephalus, generated by the hypersecretion of the choroid plexus [10,12,13,14]. This process was initially attributed to the presence of intraventricular blood components [10,15] but is now considered more of an inflammatory process triggered by a hemorrhagic event [13,16], as inflammatory markers, IL6, for example, were identified in the CSF of patients with PHVM and other various neuroinflammatory disorders [10,17,18].

The enlargement of the ventricles affects circulation in the adjacent cerebral zones, causing an ischemic process that interferes with axonal and oligodendrocyte precursor development [6,19]. PHVM disturbs the development of the premature brain through different intricate mechanisms, including mechanical distortion, subsequent ischemia, neuroinflammation, and neurotoxicity [1,20].

PHVM can evolve to a spontaneous regression, limitation, or increase in ventricular size, with the latter requiring neurosurgical intervention [19]. Traditional practice advised an expectative approach, waiting for the self-limitation of the pathologic process, considering that in the absence of high intracranial pressure (HIP), demonstrated in about one-third of patients with IVH [21], ventricular enlargement should be tolerated. More recent research proved that early interventions, before clinical signs, can limit the consequences of the pathological cascade that impairs the neurologic development of the premature brain.

### 1.3. Challenges in Management of IVH and PHVM in NEONATAL Intensive Care Units (NICUs)

Despite improvement in neonatal care, preventive medical interventions cannot always influence the incidence of IVH [22] and, therefore, neonatologists should focus on reducing the severity of the consequences: PHVM and HIP. The sooner IVH and PHVM are diagnosed, the better the chances of decreasing poor neurologic outcomes. Once PHVM is detected, ventricular size should be monitored to prevent intracranial pressure from becoming harmful. Interventions that decrease CSF accumulation (acetazolamide, loop diuretics, or lumbar puncture) can prevent progressive neuronal damage, although these are not risk-free [4]. The best management of PHVM is still a matter of debate among neonatologists, pediatric neurologists, and pediatric neurosurgeons, although there are decades of research on this subject [6]. The decision to perform surgery takes into consideration the patient’s age (gestational and postnatal), etiology, and physiopathology, imagistic findings, and associated comorbidities [21]. Protocols differ from one medical center to another, according to local expertise and resources available. While neonatologists fear the negative impact of PHVM on brain maturation and the complications of prolonged admittance in NICUs, neurosurgeons usually impose a weight threshold of around 2000 g, invoking the anesthetic risk and technical difficulties. A Cochrane meta-analysis showed that mortality rates for patients with PHVM are not significantly different with or without early surgical intervention [23]; nevertheless, neurodevelopmental scores performed in toddlers show much better cognitive and motor outcomes if PHVM has been managed with an early approach [23].

ELVIS (Early vs. Late Ventricular Intervention Study) is a landmark randomized trial comparing two groups of patients with diagnosed PHVM who underwent surgery at different grades of dilatation (a low and a high defined threshold); it has demonstrated that 2–3 temporizing LPs can lead to a reduction in the need for neurosurgery in 25% of the included cases [4,6]. Researchers have provided reference measurement values for different ventricular diameters and ratios to brain or skull dimensions that provide the basis for different local protocols [6,24].

### 1.4. Neurologic Outcomes of Babies with IVH and PHVM—Prediction and Prevention

Severe IVH and PHVM are life-threatening complications encountered in NICUs; the actual mortality rate is variable depending on the level of care in different centers (about 13–40%) [4,20]. Mortality also differs depending on severity: 4% for grade I, 10% for grade II, 18% for grade III, and 40% in the presence of PVHI [5]. Survivors are at risk of developing neurologic sequalae, the outcome being dependent on the grade of IVH and the presence of PVHI. Neurodevelopmental impairment (NDI) is encountered in at least 30% of cases [4,25], manifesting as lower cognitive and language scores, cerebral palsy, mental disabilities, and/or progressive ventriculomegaly [4,8,26].

In most cases, IVH grade I and II are usually considered “benign”, although they can interfere with typical neurodevelopment [26,27]. The risk of CP and major cognitive impairments is estimated to be between 50 and 75%, as well as 45 and 86%, in patients with grade III-IV intraventricular hemorrhage, respectively, [8,26]. The gravity of NDI is proportional with ventricular size [28,29]. The outcome is therefore influenced by the magnitude of the hemorrhagic process, which damages the anatomic structures involved, but also by the consequences of impaired cerebrospinal fluid (CSF) circulation throughout the ventricular system.

Neurologic disfunctions are proportionally more severe as the grades of IVH increase, and the diagnoses of PHVM and PHVI represent independent risk factors for delayed or impaired cognitive and motor scores in early childhood [26].

### 1.5. Role of CUS in Management of Neurologic Complications of Prematurity

In NICU, cranial ultrasonography (CUS) is a perfect tool to diagnose IVH, in order to objectively evaluate PHVM and monitor the structural changes; serial measurements of the lateral ventricles can be made, and results may be plotted on a graph. Different landmarks, diameters, and ratios define the gravity and prognosis of both IVH and PHVM [1,6,24]. IVH and ventricular dilatation are occasionally diagnosed antenatally by obstetricians; in the fetus, the assessment of ventricular size and the diagnosis of ventriculomegaly are based on the measurement of the ventricular atrium (atrial width, AW) [30]. Regrettably, AW has not been validated in the neonate, and other measurements should be evaluated for ventriculomegaly after birth.

Standard protocols in most medical centers recommend the first CUS at 48–72 h after birth and repeating it at 7 days and every other week until 40 weeks post-conception, if clinical examination does not show abnormal neurologic signs [31]. An accelerated increase in head circumference or suggestive symptomatology (apnea, tone alteration, seizures, and abnormal eye movement) can impose an ad hoc ultrasound examination. Lately, thanks to the introduction in Neonatal Intensive Care Units (NICUs) all around the world of the POCUS (point-of-care ultrasound) concept, more neonatologists perform CUS more often for the diagnosis and monitoring of IVH, outside of a strict schedule, as a completion of clinical evaluation.

Ultrasonography performed in the first day of life reveals 50% of IVH, while at 72 h of life, more than 90% of the IVH cases are diagnosed [31,32,33]. If grade I or II IVH can be deceitful for an unexperienced examiner, when we discuss grade III IVH or PHVM, there is a consensus that diagnosis is very clear and easy to grasp.

PHVM becomes apparent between 2 and 6 weeks of life and can be monitored by recording images and measuring standard distances in standard planes. There is enough evidence about what is to be evaluated; the medical literature and different experts have proposed different measurements that serve as criteria for diagnosing progressive ventriculomegaly, an “alarm” size of ventricular volume, and the rhythm of examination [31,34].

Classical protocols were focused on tracking changes in one standard measurement: the ratio between the biventricular diameter and the biparietal diameter, obtained from the anterior coronal view, at the confluence between the lateral ventricles and the third ventricle (Evans ratio). Because changes in head circumference as well as in fontanel pressure appear late in ELBW infants due to higher physiological compliance and larger extracerebral space, the Evans ratio and head circumference may lead to misinterpretation. In the past decade, experts have discovered that a better understanding of the gravity of the condition can be obtained from looking at more than one ratio.

Other standard measurements include the ventricular index (VI), also known as Levine Index; the anterior horn width (AHW); the thalamo-occipital distance (TOD); the fronto-temporal horn ratio (bifrontal diameter + bitemporal diameter/2 × biparietal diameter) (FTHR); and the fronto-occipital horn ratio (bifrontal diameter + bioccipital diameter/2 × biparietal diameter) (FOHR) [4,6,28]. Of all these measurements, the FOHR measured on CUS is consistent with the fetal AW measured in prenatal MRI [35].

**Figure 1:**
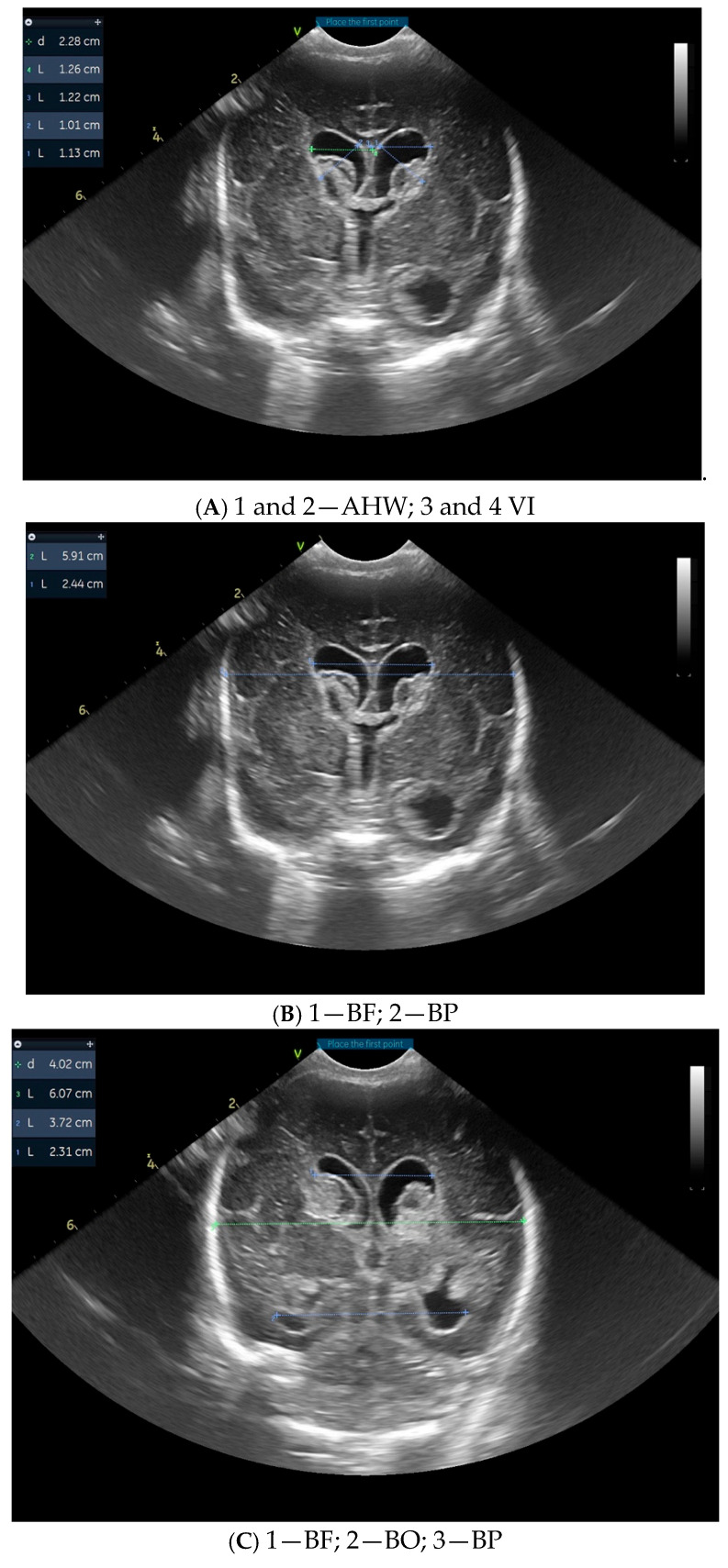

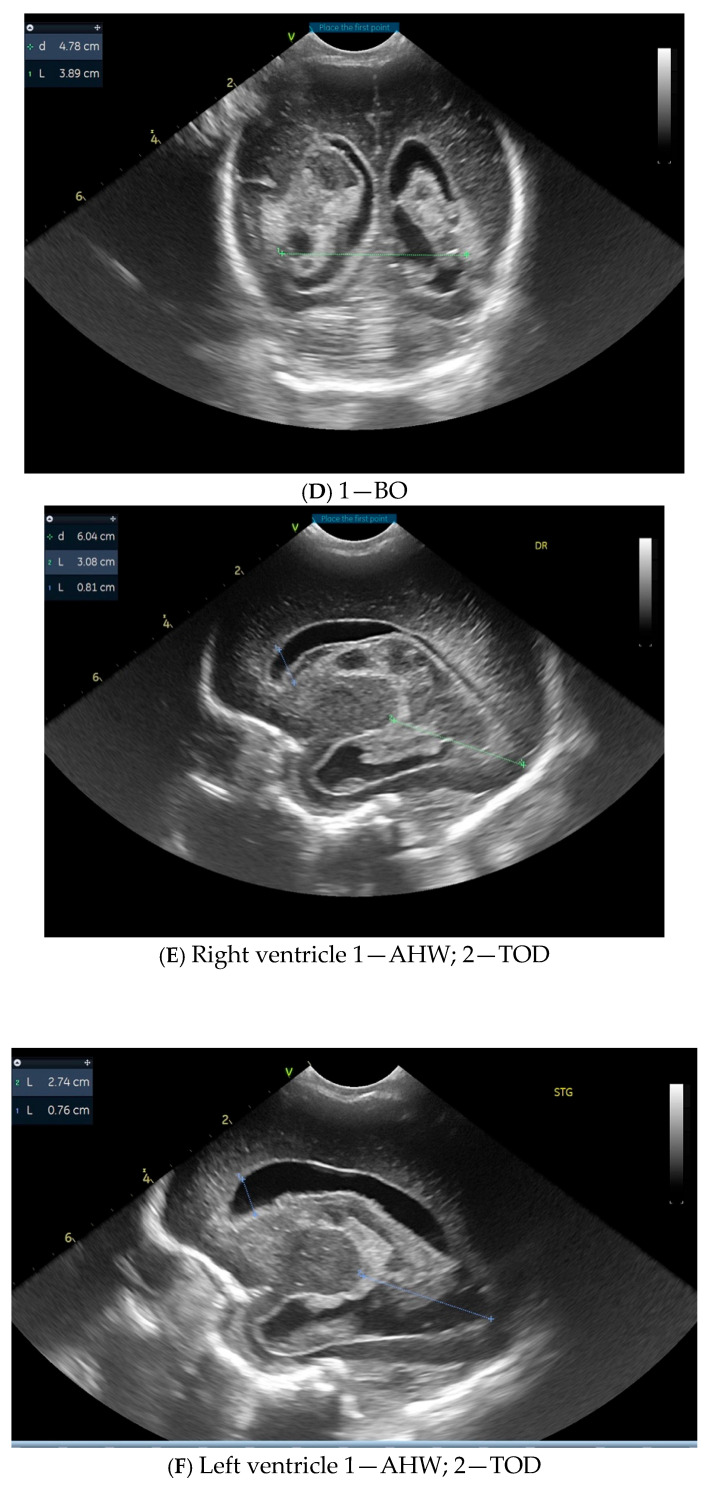
Images from cranial ultrasound of a 26 weeks of gestation preterm with different measurements. (**A**,**B**) = Coronal cUS at level of Foramen of Monro, showing extensive echogenic material within the lateral ventricles, consistent with intraventricular hemorrhage. Ventricular dilatation is also noted, reflecting post-hemorrhagic changes. (**C**,**D**) = coronal cUS at the level of occipital horn, showing the hemorrhage that extends into the adjacent periventricular white matter, visible as areas of increased echogenicity, indicating parenchymal involvement and venous infarction. (**E**,**F**) sagittal cUS with lateral ventricles that are globally enlarged and the intraventricular clot that appears as an inhomogeneous, irregular hyperechoic mass that occupies more than 50% of the ventricular volume. VI = Ventricular Index, AHW = Anterior Horn Width, TOD = Thalamo-Occipital Diameter; BF = bifrontal horn, BP = biparietal, BO =bioccipital horn.

Figure 1 illustrates cranial ultrasound images of a preterm infant born at 26 weeks of gestation, showing findings consistent with grade III intraventricular hemorrhage with periventricular infarction. There is echogenic material filling the lateral ventricles, indicating extensive intraventricular blood. In addition, increased echogenicity is seen within the periventricular white matter, suggestive of parenchymal involvement due to venous infarction. Ventricular dilatation is present, reflecting posthemorrhagic changes. Diagnosis is supported by the presence of echogenic material that occupies more than 50% of the enlarged ventricular volume and hyperechogenicity in the adjacent white matter.

These ultrasonographic findings are associated with a high risk of long-term neurologic impairment. Within four weeks, the described lesions evolved towards obstructive hydrocephalus and periventricular leukomalacia.

Since sonographic findings can predict the neurologic development of a premature infant, it is important to apply the best examination protocol to obtain accurate and comprehensive information. The objective of our analysis was to identify the sonographic criteria in the follow-up of the ventricular dilatation that describe the demand for neurosurgery and anticipate neurologic deterioration.

## 2. Methods

This study is a literature review aiming to synthesize current evidence regarding the pathophysiology, diagnosis, and monitoring of posthemorrhagic ventriculomegaly (PHVD) in preterm infants, with particular emphasis on the role of CUS as a primary imaging modality. A comprehensive literature search was conducted in the electronic databases PubMed, Scopus, and Web of Science, covering the period from January 2000 to April 2025. The search strategy used free-text terms including the following: “post-hemorrhagic ventricular dilatation” or “posthemorrhagic ventriculomegaly”, “cranial ultrasound” or “cranial ultrasonography”, “intraventricular hemorrhage”. Filters were applied to select only peer-reviewed original studies, systematic reviews, and meta-analyses.

**Figure 2:**
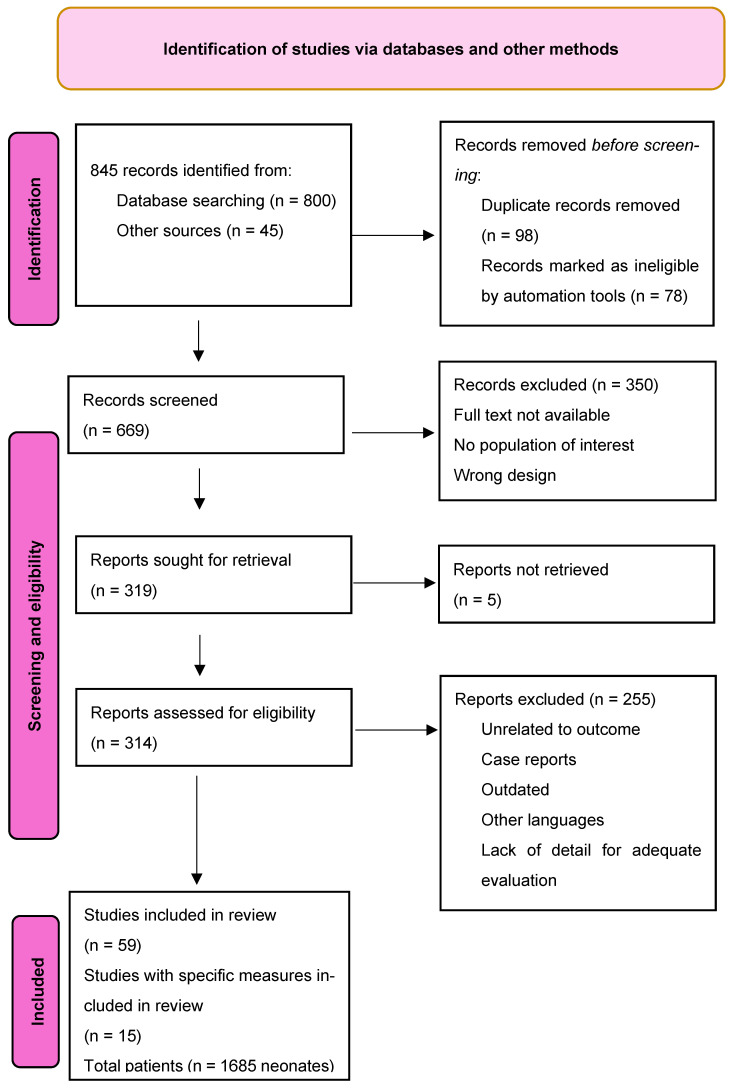
Flow diagram.

The selection process of eligible studies was carried out in accordance with the PRISMA (Preferred Reporting Items for Systematic Reviews and Meta-Analyses) guidelines. Figure 2 illustrates the detailed flow of information through the different phases of the review, including identification, screening, eligibility assessment, and final inclusion.

## 3. Results

Although CUS is standardized in most institutions, there are differences among centers in the criteria used to decide the therapeutic intervention or the referral to neurosurgery; from previous studies, we have extracted significant correlations between described examination protocols and evaluated outcomes, aiming to identify the best measurements that predict the need for surgery or problems in future neurodevelopment (Table 1). The table highlights key findings from the referenced studies, emphasizing how variations in ventricular dimensions correlate with prognostic implications across different patient populations.

## 4. Discussion

Although MRI is gaining more ground among medical investigations at present and it would be superior to CUS in detecting small intracranial hemorrhages, for LBW and ELBW infants, respiratory and hemodynamical instability are serious limiting factors for its extended use. The need for transport, high costs, and the impossibility to perform serial examinations make MRI an investigation recommended to preterm babies only at term-corrected age in most guidelines worldwide; its major advantage is detecting white matter injury and cerebellar hemorrhage [47].

Ultrasound measurements in trans-fontanel examination are easy to perform, and by using different sections and windows, it is possible to obtain a quick tridimensional picture of the brain and structural alterations. Even so, a single examination cannot represent the basis for a medical decision or intervention, while serial evaluations are more reliable, tracking a direction and reflecting an evolutive pattern [6]. Respecting a protocol, CUS becomes less operator-dependent (decreased intra- and inter-observer variability) [28].

In cases of PHVM, CUS can predict the need for surgery from early scans, but some indices seem to be more reliable than others. The ELVIS trial defined a low and a high threshold of ventricular dilatation based on VI and AHW dimensions, which became a reference for much subsequent research [19,20,36]. These most used ultrasonographic criteria for the evaluation of PHVM are the ventricular index (VI) and ventricular width over the 97th percentile for gestational age or anterior horn width (AHW) > 6 mm [19,20,48]. A study published in 2022, based on 13 years of research (2007–2020), calculated the thresholds of common ventricular indices that could predict which patients are at greater risk of receiving surgical intervention [28]; VI (ventricular index) is dependent on gestational age and is interpreted in comparison to a reference, thus being more difficult to interpret; AHW, FOHR, and FTHR reflect the severity of the pathological process, regardless of the stage of brain development. Experts have concluded that AWH is the most reliable index [28,42,43,49,50].

The Hydrocephalus Research Network (HCRN) use FOHR > 0.50 as a threshold for referral to neurosurgery and FOHR > 0.55 and onset of intracranial raised pressure as criteria for intervention; still, many institutions worldwide (including our own) initiate CSF diversion only at FOHR 0.65–0.68 [28,51,52]. On the other hand, there are medical centers that recommend CSF diversion at AHW > 6–10 mm, VI > p97–p97+4, even if the patient is asymptomatic [38].

Comparing the sensitivity and specificity of different indices studies showed that AHW measured in the first scans is best correlated with the resolution or progression of PHVM: it is demonstrated that any increase in AHW beyond 6 mm significantly reduces the probability for spontaneous resolution [19]. AHW can more accurately predict PHVM progression and neurosurgical intervention compared with VI and FTHR [41]. If PHVM is identified only on one anatomic side, there is better prognosis than the symmetrical enlargement [19].

When performing CUS, ventricular volumes are estimated best by calculating the values of FOHR and FTHR, which align closely with MRI findings. FTHR was measured in infants with grade III or PHVI, and the authors demonstrated that it correlates with lower fractional anisotropy values; moreover, in evaluating children of 1–2 years old by gross motor function classification scores, they were able to demonstrate a relationship between this ratio and neurodevelopmental status [20]. Even so, researchers consider that AHW is a better indicator of PHVM gravity [6,44]

The enlargement of the thalamo-occipital distance (TOD) is also an ultrasonographic alteration that can become visible before the enlargement of the anterior horns [19]. TOD above 25 mm is considered a threshold value for early surgical intervention [36]. Recent research included FOHR among risk criteria for functional impairment at school age, at a value of 0.61 [28,45]. Comparing inter-rater variability for AHW, VI, and FOHR, many authors consider AHW to be most consistent; because AHW is a linear measurement and okFOHR/FTHR are ratios of three diameters, it implies less subjectivity [28]. Among the difficulties in performing CUS is the poor visibility in the lateral parietal region due to the bony structure of the calvaria. Also, when intraventricular clots or porencephaly are present, it is more difficult to obtain exact measurements, since ventricular borders are less defined [28].

Although most used, VI may become inaccurate if it is not reported at the correct gestational age (GA), or if the patient is below 24 or beyond 42 weeks of gestation. References for GA are established according to single-center measurements and therefore cannot be considered generally valid [24,28]. As FOHR and FTHR are not influenced by GA and reflect the tri-dimensional structure of the ventricular system, these measurements are useful in addition to AHW and VI [28].

**Figure 3:**
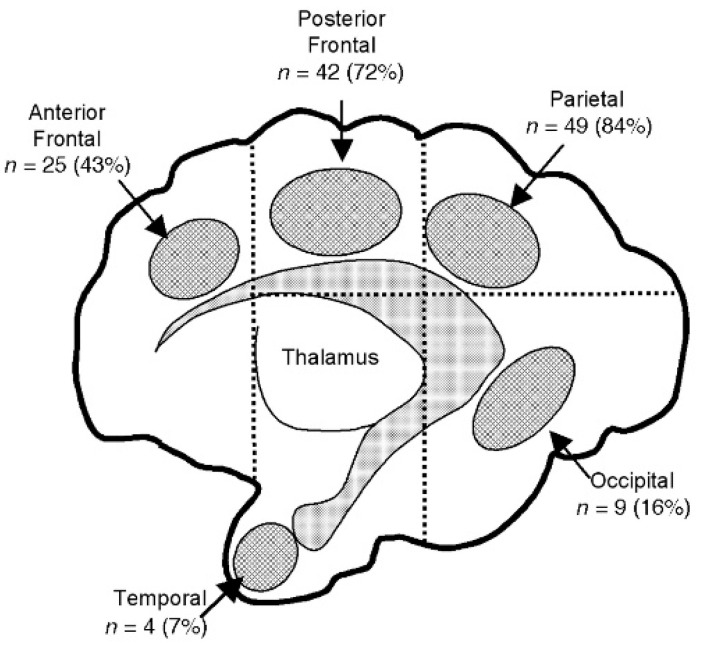
Sagittal plane that illustrates the graphical scoring made by Bassan.

For patients with IVH or PHVM and PHVI, the Bassan classification describes more detailed features of intra-parenchymatous lesions: after dividing the brain into five zones by two perpendicular lines tangent to the thalamus, as seen in Figure 3, the frontal anterior, frontal posterior, parietal, occipital, and temporal areas are evaluated using three criteria, noted with 0 or 1: localization (single = 0; multiple lesions = 1), laterality (unilateral = 0 or bilateral = 1), and midline shifting of the anatomic structures (no = 0; yes = 1) [53].

Although less used in practice, this subclassification of PVHI is more precise and helps clinicians to predict more accurate future neurologic impairment; according to this criterion, a patient with PHVI scoring 0 can provide a normal motor examination at 12 months of age [53].

Another advantage of CUS is that it can provide supplemental information regarding intracranial pressure by assessing the resistive index (RI), with Doppler US; with progressive PHVM, systolic flow in cerebral circulation initially rises and subsequently decreases, as intracranial pressure rises. These changes can be simulated by compressing the anterior fontanel to evaluate cerebral compliance [6].

In search for the best therapeutic approach in infants with ventriculomegaly when a neonatologist has to decide to initiate lumbar punctures or to refer the case to neurosurgery, in this meta-analysis, we attempted to find out the advantages and disadvantages of different measurements and to evaluate the utility or futility of multiple measurements in the same patient. Table 2 provides a comparative analysis of the most used measurements in PHVM to enhance interpretability and limitations, according to different authors.

When evaluating PHVM, researchers also looked for the most reliable time of measurement of the above-mentioned indices, and comparing size deviation at serial CUS, they discovered that early ventriculomegaly (in the first 2 weeks of life) may be associated with a higher risk of progressive and persistent ventriculomegaly [27,38]. Half of the infants who experienced spontaneous resolution had resolutions within 14 days after PHVM diagnosis and 71% within 21 days [19,27]. The number of CUS reviewed per patient can reach 9–10 per patient from diagnosis to stabilization or referral to neurosurgery [19].

Neonatologists and neurosurgeons worldwide are concerned about the best timing and most reliable criteria for surgical intervention, in order to balance the risk and the benefits. Although studies have established thresholds for different interventions, local expertise and resources make a difference in the management of cases. Neonatologists should record in a graphical manner the progression of ventricular volume, using all indices described (VI, AHW, TOD, FOHR, and FTHR), as each one of them reflects a different perspective. Reporting values to reference for age and to previous evaluations is necessary and usually required by the neurosurgeon.

The most significant factor driving the timing of neurosurgical intervention seems to be the medical center where patients were treated [6,56]. It is important to acknowledge that according to available real-world data, there are patients with ventricular size over the high threshold established by the ELVIS trial who also had spontaneous resolution, without major NDI. Therefore, adopting the low threshold for neurosurgical intervention for all newborns with PHVM could lead to potentially unnecessary interventions [19,20,36]. Understanding the dynamic of ventricular volume and continuous ultrasonographic and clinical monitoring is essential for planning surgical interventions.

## 5. Conclusions

There is a clear advantage in performing serial CUS supplementary to the routine weekly protocol in the case of VLBW and ELBW neonates with IVH. Bedside and on-demand examination by the neonatologist improves the timing of diagnosis, the accuracy of staging, and adequate management. Since 1970, when CUS was first used in neonatal care, the method has been constantly improved, both from a technological point of view (better machines with higher resolution and faster processing) and from operator-dependent factors (use of extended protocols and better interpretation) [31,57,58]. Currently, it is commonly accepted that the accuracy of ultrasonographic investigation is superposable for many structural abnormalities with magnetic resonance imaging (MRI) [31]. Easier to perform, rapidly available, less expensive, and less stressful for the patient, it is superior to other imagistic exams due to the possibility of repeated real-time interrogation. Different acoustic windows, frequencies, and modes provide a comprehensive picture of different brain pathologies; monitoring PHVM is a worthwhile and valuable approach that allows earlier interventions and can alleviate developmental impairments. Scanning at term-equivalent age for premature neonates reveals the actual state of brain maturation and residuals of gray and white matter injuries, making CUS a valuable tool for assessing neurologic prognosis, with a predictive value comparable to that of conventional MRI [57,59]. Using evidence-based protocols, the acknowledgement of advantages and limits of different measurements improves clinical judgment and leads to optimal interventions. By routinely using CUS, neonatologists can not only diagnose the condition but also objectively and dynamically quantify structural alterations. The values of VI, AHW, FOHR, and FTHR exceed the thresholds, and the onset and progression of ventricular dilatation are important and play a more decisive role in leading medical conduct for patients with IVH and PHVM.

Future developments in neonatology will involve technological progress put into the service of the clinicians; along with solid theoretical knowledge and practical bedside experience, CUS will remain a reliable tool able to improve medical decisions. From birth to discharge, all medical care should be guided by evidence-based and safe interventions, aiming at the best possible long-term developmental outcomes.

## Figures and Tables

**Table 1:** Relationship between ventricular measurements for PHVM and main outcomes and conclusions in referenced studies.

**Index/Ratios with Threshold Values**	**Study—Name/Type**	**Population**	**Outcomes**	**Conclusions**
Low threshold (LT) VI > p97, AHW > 6 mm, FOHR—430.43 High threshold (HT) VI = p97 + 4 mm, AHW > 10 mm, FOHR—0.49	ELVIS (European Early vs. Late Ventricular Intervention Study, 2002–2007); multicenter retrospective study (five NICUs) “Treatment thresholds for intervention in posthaemorrhagic ventricular dilation: a randomised controlled trial” [36]	95 patients with PHVM; 31 treated at LT; 42 infants treated at HT 22 patients without intervention	Correlation between time of onset of treatment of PHVD and difference in the requirement of a ventriculoperitoneal (VP) shunt and/or neurodevelopmental outcome	Late intervention required shunt insertion significantly more often than those treated early.
Low threshold VI > p97, AHW > 6 mm, FOHR—0.43 High threshold VI = p97 + 4 mm, AHW > 10 mm Ventr. volume	“Ultrasonographic Estimation of Ventricular Volume in Infants Born Preterm with Posthemorrhagic Ventricular Dilatation: A Nested Substudy of the Randomized Controlled Early Versus Late Ventricular Intervention Study (ELVIS) Trial” [37]	59 patients from ELVIS from four participating centers	Correlation between ventricular volume (VV) in PHVM and prediction for ventriculoperitoneal (VP)-shunt insertion and 2-year or NDI	Maximum VI and AHW before reservoir insertion were independently associated with the need for VP shunt. The proposed thresholds in the ELVIS trial were associated with significant different long-term outcomes.
Low threshold VI > p97, AHW > 6 mm, FOHR 0.42 Hgh Threshold VI = p97 + 4 mm, AHW > 10 mm, FOHR 0.48	“Assessment of Brain Injury and Brain Volumes after Posthemorrhagic Ventricular Dilatation: A Nested Substudy of the Randomized Controlled ELVIS Trial” [38]	126 preterm infants ≤ 34 weeks and PHVM	Kidokoro Global Brain Abnormality Score and the frontal and occipital horn ratio were measured	FOHR was lower in the low-threshold group (*p* = 0.001). Infants in the low-threshold group had a normal or mildly increased score vs. infants in the high-threshold group (*p* = 0.002).
VI minimum p97 + 4 mm, VI max—no upper limit	“Drainage, irrigation and fibrinolytic therapy (DRIFT) for posthaemorrhagic ventricular dilatation): 10-year follow-up of a randomised controlled trial” (four centers) [39]	77 preterm infants with IVH and progressive PHVM	Death Risk of VP shunt NDI	No significant difference in terms of death or need for VP shunt, but significantly lower NDI in the treatment arm.
Low threshold VI > p97 AHW > 6 mm TOD > 25 mm High threshold p97 + 4 mm AHW > 10 mm	“Treatment thresholds for intervention in posthaemorrhagic ventricular dilation: a randomised controlled trial” [36]	126 preterm < 34 weeks (14 NICUs)	Death Risk of VP shunt NDI	There was no significant difference in the primary composite outcome of VP shunt placement or death or lower VP shunt rate. Infants treated at the lower threshold received more invasive procedures.
FOHR 0.66 FTHR 0.62 AHW 15.5 mm VI 8.4 mm > p97	“Ventriculomegaly thresholds for prediction of symptomatic posthemorrhagic venticular dilatation in preterm infants” [28]	132 patients Retrospective, 2007–2020	Threshold values of ventricular indices predicting surgical intervention	Indices were predictive from the first scans for progressive persistent ventriculomegaly with different sensitivity and specificity.
VI > p97, AHW > 6 mm (low threshold) VI > p97 + 4 mm and AHW > 10 mm (high threshold)	“Randomized Controlled Early versus Late Ventricular Intervention Study in Posthemorrhagic Ventricular Dilatation: Outcome at 2 years” [40]	126 infants	Death or cerebral palsy or Bayley composite cognitive/motor scores < −2SD at 24 months corrected age	Earlier intervention was associated with lower odds of death or severe NDI.
FOHR > 0.55	“Neurodevelopmental outcomes of permanent and temporary CSF diversion in posthemorrhagic hydrocephalus: a Hydrocephalus Clinical Research Network study” [29]	106 patients 2012–2021	Neurodevelopmental outcomes in preterm infants diagnosed with PHVM after temporary vs. permanent CSF diversion strategies	NDI is correlated with high values of FOHR at conversion to permanent diversion.
Early approach (EA) VI < +2SD AHW < 6 mm Late approach (LA)	“Posthemorrhagic ventricular dilatation in preterm infants: When best to intervene?” [41]	127 preterm infants, gestation < 30 weeks, with early vs. late approach	Death Risk of VP shunt NDI	Significant lower rate of shunt placement in EA group. In the LA group, survivors had lower cognitive and motor scores (*p* = 0.002); VI comparable in EA/LA groups; AHW larger in infants undergoing intervention (*p* = 0.04).
VI AHW FHOR	“Post-hemorrhagic ventricular dilatation: inter-observer reliability of ventricular size measurements in extremely preterm infants” [42]	139 preterm infants with IVH	Inter-observer reliability of these indices for prediction of severe PHVD	AHW and VI are highly reproducible in experienced hands compared to FTHR, with AHW from the second week onwards being the strongest predictor for receiving surgical intervention.
VI > p97 AHW > 6 mm compared to VI and FOHR	“Spontaneous resolution of post-hemorrhagic ventricular dilatation in preterm newborns and neurodevelopment” [19]	88 preterms with PHVM MCT retrospective study, 2007–2020	Role of ventricular indices in prediction of spontaneous resolution of PHVM and NDI	Spontaneous resolution of PHVM was better predicted by smaller AHW. Subjects with spontaneous resolution of PHVM had significant lower NDI.
FTHR 0.51	“The utility of the fronto-temporal horn ratio on cranial ultrasound in premature newborns: a ventriculomegaly marker” [43]	100 neonates with IVH; retrospective study—2011–2014	Identifying the normal value of FTHR on CUS in relation to WMI and cerebral palsy (CP)	The FTHR cut-off point of 0.51 had the highest sensitivity and specificity for moderate-to-severe WMI. In the IVH grade 3–4 group, the elevated FTHR correlated with a lower FA and higher GMFCS.
FTHR FOHR	“Frontal occipital and frontal temporal horn ratios: Comparison and validation of head ultrasound-derived indexes with MRI and ventricular volumes in infantile ventriculomegaly” [44]	90 infants < 6 months of age with PHVM 3-year retrospective study	Assessment of FOHR and FTHR obtained from cranial ultrasound as reliable measures of ventriculomegaly in infants	FOHR and FTHR obtained from CUS have excellent inter-observer concordance, are concordant with MRI-derived linear ratios, and correlate with MRI-derived ventricular volumes. US-derived FOHR and FTHR are reliable indexes for clinical follow-up of PHVM.
FOHR 0.62 ± 0.12 at surgical consult and 0.75 ± 0.13 at intervention (*p* < 0.001)	“Degree of ventriculomegaly predicts school-aged functional outcomes in preterm infants with intraventricular hemorrhage” [45]	134 infants with Grade III/IV IVH (Papile)	NDI for patients with PHVM	Ventriculomegaly measured by FOHR and PVL are independent correlates of school-age functional outcomes in preterm infants with IVH regardless of need for neurosurgical intervention.
Longest diagonal diameter of the PHVI Localization, shape, and midline shifting were noted (0–3)	“Periventricular Hemorrhagic Infarction in Very Preterm Infants: Characteristic Sonographic Findings and Association with Neurodevelopmental Outcome at Age 2 Years” [46]	160 infants with median age 26.6 weeks and IVH grade III associated with PVHI	Mortality Cognitive and gross motor development	Increasing PHVI size and severity score for PHVI according to Bassan criteria are predictive for less optimal gross motor outcome and death. Ventricular dilatation is an independent risk factor for poorer cognitive and motor function.

PHVM—posthemorrhagic ventriculomegaly; VI—ventricular index; AHW—anterior horn width; FOHR—fronto-occipital horn ratio; FTHR—fronto-temporal horn ratio; MRI—magnetic resonance imaging; NDI—neurodevelopmental impairment; PHVI—posthemorrhagic venous infarction; WMI—white matter injury; GMFCS—gross motor function classification system; CSF—cerebrospinal fluid; VP shunt—ventriculoperitoneal shunt.

**Table 2:** Strengths and limitation of ventricular measurements in PHVM according to different authors.

Index/ Ratios	Refference Studies	Advantages	Disadvantages
VI	“Treatment thresholds for intervention in posthaemorrhagic ventricular dilation: A randomised controlled trial” [36] “Ventriculomegaly thresholds for prediction of symptomatic post-hemorrhagic ventricular dilatation in preterm infants” [28]	Most used in all studies [36] Best specificity [28]	Dependent on gestational age [24,54] Restricted to 24–42 weeks post-conceptionally [28] Single-institution data reference [28] Inter-rater variability (use average of both sides) [24] Intraventricular clot or periventricular infarction can make ventricular border less defined [28]
AHW	“New reference values for the neonatal cerebral ventricles” [24] “Inclusion of extremes of prematurity in ventricular index centile charts” [54] “Post-hemorrhagic ventricular dilatation: inter-observer reliability of ventricular size measurements in extremely preterm infants” [42] “Timing of Intervention for Posthemorrhagic Ventricular Dilatation: An Ongoing Debate” [20]	Independent of gestational age [24,54] Best inter-rater reliability > than VI, FOHR, and FTHR [42] Better predictor for severe PHVM compared to other indices [55]	In cases of porencephalic cysts contiguous with the ventricle, it may be difficult to measure accurately [28] Intraventricular clot or periventricular infarction can make ventricular border less defined [49]
FTHR/FOHR	“Optical Detection of Intracranial Pressure and Perfusion Changes in Neonates with Hydrocephalus” [55] “Fronto-temporal horn ratio: yet another marker of ventriculomegaly?” [49] “Frontal occipital and frontal temporal horn ratios: Comparison and validation of head ultrasound-derived indexes with MRI and ventricular volumes in infantile ventriculomegaly” [44]	Independent of gestational age [24,54] Higher sensitivity compared to VI and AHW [28] Good correlation with ventricular volume measured from MRI [44] Reflects better ventricular size and injury of white matter (subsequently with NDI) [44]	The lateral boundaries of the parietal skull can be obscured, making measurement difficult Not correlated with high intracranial pressure [20,49] Lower inter-rater reliability compared to VI and AHW (requires three measurements) [28]

VI—ventricular index; AHW—anterior horn width; FOHR—fronto-occipital horn ratio; FTHR—fronto-temporal horn ratio; MRI—magnetic resonance imaging; NDI—neurodevelopmental impairment.

## Data Availability

The original contributions presented in this study are included in the article. Further inquiries can be directed to the corresponding author.

## Abbreviations

AHW—anterior horn width; AMPA—α-amino-3-hidroxi-5-metil-4-isoxazolepropionic acid; BF—bifrontal; BO—bioccipital; BP—biparietal; COX—cyclooxygenase; CP—cerebral palsy; CSF—cerebrospinal fluid; CUS—cranial ultrasound; EA—early approach; EGF—epidermal growth factor; ELBW—extremely low birth weight; ELVIS—European Early versus Late Ventricular Intervention Study; FOHR—fronto-occipital horn ratio; FTHR—fronto-temporal horn ratio; GA—gestational age; GMFCS—gross motor functional classification system; HIP—high intracranial pressure; HCRN—hydrocephalus research network; IVH—intraventricular hemorrhage; IL6—interleukin 6; LA—late approach; LBW—low birth weight; LP—lumbar puncture; MRI—magnetic resonance imaging; NDI—neurodevelopmental impairments; NFKB—nuclear factor kappa-light-chain-enhancer of activated B cells (NF-kB); NICU—neonatal intensive care unit; PAR1—protease -activated-receptor; PHVI—posthemorrhagic venous infarction; PHVM—post hemorrhaging ventriculomegaly; POCUS—point-of-care ultrasound; RI—resistive index; TLR4—Toll-like receptor 4; TRPV—transient receptor potential cation channel subfamily V; VI—ventricular index; VP—ventriculoperitoneal; VV—ventricular volume; WMI—white matter injury.
